# Neuro-pharmacological effects of *Crinum zeylanicum *in mice

**Published:** 2012

**Authors:** Adeniyi Yahaya Tijani, Oluwakanyinsola Adeola Salawu, Good-luck Jaiyeoba, Joseph Akponso Anuka, Isah Marte Hussaini

**Affiliations:** 1 *Department of Pharmacology and Toxicology, National Institute for Pharmaceutical Research and Development Idu industrial Area, Abuja, Nigeria*; 2 *Department of Pharmacology and Therapeutics, Faculty of Pharmaceutical Sciences, Ahmadu Bello University, Zaria, Nigeria*

**Keywords:** *Crinum Zeylanicum*, Pentobarbitone, Psychosis, Sleep, Stereotype Behaviour

## Abstract

**Objectives**: The aim of present study was to evaluate some effects of *Crinum zeylanicum* (*C. zeylanicum*) on central nervous system.

**Materials and Methods**: *C. zeylanicum* methanolic bulb extract (250-1000 mg/kg orally), 2 mg chlorpromazine and 4 mg diazepam /kg body weight intraperitoneally respectively were tested in mice using Irwin test, pentobarbitone-induced sleep test, spontaneous motor activity, apomorphine-induced stereotype behaviour, and rota-rod performance.

**Results**: The *C.* *zeylanicum* bulb extract significantly and dose-dependently decreased apomorphine-induced hyperactivity in mice (p<0.001). The Irwin test revealed dose-dependent central depressant effect of the extract, shortened (p<0.05-0.01) the onset of sleep and prolonged the duration of sleep. The extract produced significant (p<0.05-0.001) and dose- dependent reduction in spontaneous motor activity and apomorphine-induced stereotype behaviours in mice. The extract had no effect on performance of mice on rotarod.

**Conclusion**: The results suggest that the extract may possess sedative principles with potential neuroleptic properties.

## Introduction

Interest in the use of medicinal plants to treat various central nervous disorders including depression, epilepsy, and psychosis is on the increase worldwide (Kinjal Chauhan et al., 2011 ▶). Plant kingdom has remained a target of search for new drugs and lead compounds by multinational drug companies and research institutes (Mahendran et al., 2011 ▶). The World Health Organization (WHO) supported programs designed to use medicinal plants more effectively in traditional health care systems especially in developing countries (WHO, 2000 ▶) where they are readily available, easily affordable, and already integrated into the people’s cultures.* Crinum zeylanicum* Linn. (*C. zeylanicum*), (Family: Amaryllidaceae) is one of such medicinal plants used in traditional treatment of ailments. 

It is a bulbous plant that is widely distributed in tropical Africa. In western part of Nigeria, the bulb is used externally for skin troubles, injuries, and on refractory ulcers (Adesanya, 1992 ▶). In southern part of Nigeria, the Ibinis use juice obtained from the bulb for management of general debility, childhood convulsions, epilepsy, and psychosis (Jayeoba, personal communication). Previous study in our laboratory has shown that methanolic bulb extract possesses anticonvulsant effect against leptazol-induced convulsion in mice (Tijani et al., 2010 ▶). The objective of this study was to evaluate the possible neuroleptic potential of this widely used plant in the management of neuropsychiatric disorders.

## Materials and Methods

**Drugs and chemicals**

Pentobarbitone sodium (Sigma chemical Co., USA), diazepam (Sigma chemical Co., USA), chlorpromazine (Sigma chemical Co., USA), and apomorphine (Sigma chemical Co., USA)

**Plant material**

The whole plant (bulb, leaves, and flower) of *C. zeylanicum* was collected by Mr. Goodluck Jaiyeoba, a traditional herbal medicine practitioner from Rafin Sayan, a village in Suleja, Niger state of Nigeria. The plant was identified and authenticated by Mrs. Jemilat Ibrahim, a taxonomist with the Department of Medicinal Plant Research and Traditional Medicine, National Institute for Pharmaceutical Research and Development (NIPRD), Idu-Abuja where a voucher sample (NIPRD/H/6258) was prepared and deposited.

**Extraction of plant material**

The bulb of *C. zeylanicum* was crushed and air-dried at room temperature. One hundred grams (100 g) of the dry plant material was macerated in 70% methanol for 48 hours. The resulting mixture was filtered using muslin cloth followed by Whatman filter paper (No. 1). The aliquots obtained was dried on water bath and stored at -4 °C until required for use.

**Animals**

Albino mice (18-20 g) of both sexes obtained from the Animal Facility Centre of National Institute for Pharmaceutical Research and Development (NIPRD), Abuja, Nigeria were used in the study. The mice were fed standard laboratory diet, given water *ad libitum *and maintained under laboratory conditions of temperature (22±1 °C), relative humidity (14±1%) and 12 h light and 12 h dark cycle. All experiments were performed between 7 and 11 AM in accordance to the “Guide for the Care and Use of Laboratory Animals as adopted by the National Institutes of Health and NIPRD Standard Operating Procedures. 

**Gross behavioural effects of** ***C. zeylanicum*** **methanolic bulb extract in mice**

This study was carried out according to the method described by Irwin (1968) ▶ and modified by Perez-Saad and Buznego (2008) ▶. Adult mice of both sexes were randomized into five groups of five mice each. Group I mice received 10 ml distilled water/kg body weight. Mice in groups II, III, IV, and V received 100, 250, 500, and 1000 mg extract/kg body weight orally, respectively. The mice were placed in separate transparent cages after one hour of extract administration and were observed behaviourally for a period of one hour; (i) Central nervous system (CNS) stimulant effects such as excessive jumping, biting, sniffing, scratching, etc., (ii) CNS depressant effects indicated by excessive reduced motor activity reduced startle response and reduced response to manual manipulation, and (iii) autonomic effect such as pupillary size, lacrymation, salivation, defecation, and urination were also observed. 

**Pentobarbitone - induced hypnosis in mice**

The method of Rolland et al. (1991) ▶ was used. Mice were randomized into five groups of six mice each. Group I mice received 10 ml distilled water/kg body weight orally while those in groups II, III and IV were given 250, 500, and 1000 mg extract/kg body weight, respectively orally. Mice in group V received 2 mg diazepam/kg body weight intraperitoneally. One hour after extract and thirty minutes after diazepam administration respectively, 25 mg pentobarbitone sodium/kg body weight was administered to each mouse intraperitoneally. Each mouse was placed individually in a transparent cage and then observed for onset and duration of sleep, with the criterion for sleep being loss of right reflex on all four limbs after being gently rolled sideways. The interval between loss and recovery of righting reflex was used as the index of hypnotic effect (duration of sleep) (Ramirez et al., 1998 ▶).

**Studies on spontaneous motor activity**

The study was carried out using the method described by Amos et al., (2001) ▶. Adult mice of both sexes were randomized into five groups of six mice each. Groups I served as the control and received 10 ml distilled water/kg body weight orally. Groups II, III, and IV received 250, 500, and 1000 mg extract/kg body weight orally, respectively. The group V mice received 2 mg chlorpromazine/kg body weight intraperitoneally. One hour and thirty minutes after extract and chlorpromazine administration, the mice were transferred individually into Letical activity cages (LE 3806) consisting of four ventilated motor cages connected to a multi-counter. Activities were automatically recorded after a 1-min latency period for 6 min at 30 min intervals for a period of 120 min.

Test for motor co-ordinationRotarod Test

The study was carried out according to the method described by Perez et al., (1998) ▶. Rota rod treadmill device (Ugo Basile no. 7680, Italy) was used for this experiment. Mice trained to remain on slowly moving (16 rpm) rods of 5 cm diameter for 180 seconds or longer were selected and randomised into four groups of six mice each. Group I mice received 10 ml distilled water/kg orally. Groups II, III, and IV received 250, 500, and 1000 mg extract/kg orally, respectively. One hour after administration of extract, the mice were placed singly on the rod for 3 minutes, at 30 minutes intervals for 3 h. If an animal failed more than once to remain on the rod for 3 minutes, the test was considered positive, meaning that there is lack of motor co-ordination.

Apomorphine –induced stereotype behavioural studies in mice

The method described by Randrup and Munkvad (1967) was used for the stereotype behavioural studies. Adult mice were randomized into five groups of six mice each. Group I received 10 ml normal saline/kg body weight orally. Groups II, III, and IV received 250, 500, and 1000 mg extract/kg body weight orally, respectively. The group V mice were given 2 mg chlorpromazine/kg body weight intraperitoneally. One hour after administration of saline and extract and thirty minutes after chlorpromazine administration, all the mice were given 2 mg apomorphine/kg intraperitoneally. The signs of stereotype behaviour that included circling, jumping, sniffing, and general locomotion were recorded for a period of 2 h using a hand held tally counter (Irwin, 1968 ▶). 

**Statistical analysis**

All the data were expressed as mean±SEM. Differences in means were estimated by means of ANOVA followed by Dunnet's post hoc test. Results were considered significant at p<0.05.

## Results

**Effect of** ***C. zeylanicum*** **bulb extract on Irwin test**

The extract produced dose-dependent decrease in motor activity. Other effects observed were paw licking, erect fur, salivation, urination, and defecation (Table 1).

**Effect of** ***C. zeylanicum*** **bulb extract on pentobarbitone-induced sleep test in mice**

The extract (500 and 1000 mg/kg) significantly (p<0.05 and p<0.01) reduced the onset and prolonged duration of sleep induced by pentobarbitone. The effects are comparable to that of 2 mg diazepam/kg body weight (Table 2). 

**Table 1:** Effect of methanolic bulb extract of *C. zeylanicum* on Gross behaviour of mice

**Treatment**		**Control**	**100 mg extract/kg**	**250 mg extract/kg**	**500 mg extract/kg**	**1000 mg extract/kg**
**Autonomic effects**		None	None	Mild paw licking, erect fur	Moderate paw-licking, salivation, urination defecation	Intense paw-licking, salivation, urination defecation
**Central nervous effects**		None	None	Slight reduction in motor activity	Moderate reduction in motor activity	Significant reduction in motor activity, sedation
**Death**	None		None	None	None	None

**Table 2:** Effect of *C. zeylanicum* on pentobarbitone-induced sleeping time in mice

**Treatment**	**On-set of sleep (min)**	**Duration of sleep (min)**
25 mg pentobarbitone/kg	11.29±0.81	42.60±3.52
250 mgextract/kg±25 mg pentobarbitone/kg	11.50±1.92	43.36±2.02
500 mg extract/kg±25 mg pentobarbitone/kg	7.88±1.01*	79.80±5.37*
1000 mg extract/kg±25 mg pentobarbitone/kg	3.57±0.47**	107.60±10.69**
2 mgdiazepam/kg+25 mg pentobarbitone/kg	2.80±0.37**	113.40±4.52**

* Significantly different from the control at p<0.05, and

** at p<0.01, n= 6

**Effect of** ***C. zeylanicum*** **bulb extract on spontaneous motor activity in mice**

The extract (250-1000 mg/kg) produced significant (p<0.05 and p<0.01) decrease in spontaneous motor activity in the mice at all time intervals (Table 3). These effects were dose- and time-dependent.

**Effect of** ***C. zeylanicum*** **bulb extract on motor co-ordination (Rota-rod) in mice**

The extract-treated mice were able to maintain their posture on the rotating rod without falling for over 180 seconds and the cut-off time on the tread mill at all doses was used.

**Effect of** ***C. zeylanicum*** **bulb extract on apomorphine-induced stereotype behaviour in mice**

The extract (250–1000 mg/kg, p.o.) significantly (p<0*.*001) attenuated apomorphine -induced stereotyped behaviour in mice dose-dependently. This effect at 1000 mg extract/kg body weight was comparable to 2 mg chlorpromazine/kg body weight (Table 4)

**Table 3:** Effect of the extract on spontaneous motor activity in mice

**Time/min**	**Control**	**250 mg /kg**	**500 mg /kg**	**1000 mg/kg**	**2 mg CPZ/kg**
**0**	403.00±1.33	419.00±1.76	406.00±1.56	482.00±11.67	439.50±1.41
**30**	413.00±1.67	385.00±2.76*	313.00±1.78*	395.00±3.73*	398.50±1.78*
**60**	420.00±2.09	380.00±2.06*	308.00±1.65**	298.00±2.06**	182.70±9.99**
**90**	435.00 ± 1.52	376.00 ±2.48*	305.00± 1.52**	281.00 ± 3.06**	135.00 ±1.60**
**120**	445.00 ± 1.76	367.00 ± 2.57*	296.00 ± 1.59**	276.00 ±2.17**	106.30±8.20**

* Significantly different from the control at p≤0.05, and

** at p≤0.01, n=6

**Table 4:** Effect of *C. zeylanicum* methanolic bulb extract on apomorphine-induced stereotype behaviour in mice

**Stereotype behaviour**	**Control**	**250 mg/kg**	**500 mg/kg**	**1000 mg/kg**	**2 mg CPZ/kg**
**Sniffing**	1168.00±17.00	966.00±6.50***	810.00±4.80 ***	694.00±3.80 ***	667.00±14.00 ***
**Jumping/Climbing**	543.30±16.06	404.00±1.71***	395.80±1.80 ***	382.00±3.27 ***	369.30±3.18 ***
**Limb Licking**	502.00±1.90	478.00±3.70 ***	462.00±2.50 ***	417.00±4.60 ***	347.00±19.00 ***
**Cycling**	319.00±1.50	284.00±4.20 ***	275.00±5.40 ***	269.00±5.50 ***	264.00±5.70 ***

*** Highly significantly different from the control at p<0.001, n=6, CPZ: Chlorpromazine

## Discussion

The present study reports some neuropharmacological activities of methanolic bulb extract of *C. zeylanicum* in mice. The extract was found to produce alteration in general behavioural pattern, shortened onset of pentobarbitone-induced sleep, prolonged duration of pentobarbitone-induced sleeping, significant reduction of spontaneous motor motility, and apomorphine-induced stereotype behaviour in mice. It does not have any effect on the motor coordination. 

The present findings suggest that *C. zeylanicum* possesses CNS-depressant action. The extract reduced spontaneous motor activity. The spontaneous motor activity (SMA) is a measure of the gross motor activity of the animal, and reflects the integrity of the entire neuromuscular system and its control and regulation by the central nervous system (Parshad et al., 1997 ▶). The SMA is used to evaluate gross behavioural effects of drugs in laboratory animals (Carpendo et al., 1994 ▶). It measures the level of excitability of the central nervous system (Mansur et al., 1971 ▶) which correlates well with drug effect in humans. Any agent that suppresses SMA may possess central sedative properties (Ozturk et al., 1996 ▶). Central dopaminergic mechanisms play important roles in the control of motor activity and mental functions (Costal et al., 1989 ▶) such that the reduction in SMA produced by the methanolic bulb extract of *C. zeylanicum *at the doses used may be due to its inhibitory actions on central dopaminergic systems. Many groups of psychotropic agents including antipsychotics (Baldessarini, 1996 ▶), anticonvulsants (McNamara, 1996), antidepressants (Lowe et al., 1978), and narcotic analgesics (Reisine & Pasternak, 1996), can diminish SMA in all species of animals including humans. The ability of the extract to suppress SMA, shorten the onset and prolonged the duration of pentobarbitone–induced sleep in mice therefore suggests that it contains active principles that are sedative in nature.

The lack of inhibitory effect of the extract on motor co-ordination as observed in the treadmill suggests that the extract may not be acting via peripheral neuromuscular blockade but rather centrally thus confirming its central sedative property (Capaso et al., 1996 ▶).

The extract produced significant dose-dependent reduction in apomorphine-induced stereotype behaviour in mice. Apomorphine is an agonist at the dopamine receptor; it binds to D_2_ receptor subtype resulting in inhibition of adenylyl cyclase which reduces potassium ion conductance, and enhances calcium ion channel activity with resulting hyperactivity of dopamine in the nigro-striatal pathway. Agents that inhibit apomorphine-induced stereotypy can antagonise dopamine receptors in the nigrostriatal system (Tarsy & Baldessarini, 1986 ▶). 

The reduction in apomorphine–induced stereotype behaviour suggests that the extract effect may have been mediated via inhibition of dopaminergic system which may be correlated with its neuroleptic potential. It may be concluded that *C. zeylanicum methanolic *bulb extract contains psychoactive principles that are sedative in nature with possible neuroleptic potentials. Further studies are planned to establish mechanism of CNS-depressant action of *C. zeylanicum* by using various specific agonists and antagonists.
